# Construction of VSVΔ51M oncolytic virus expressing human interleukin-12

**DOI:** 10.3389/fmolb.2023.1190669

**Published:** 2023-05-15

**Authors:** Rwaa H. Abdulal, Jana S. Malki, Ezdehar Ghazal, Ahdab A. Alsaieedi, Sarah A. Almahboub, Muhammad Yasir Khan, Reem M. Alsulaiman, Mazen M. Ghaith, Turki S. Abujamel, Magdah Ganash, Ahmad Bakur Mahmoud, Almohanad A. Alkayyal, Anwar M. Hashem

**Affiliations:** ^1^ Vaccines and Immunotherapy Unit, King Fahd Medical Research Center, King Abdulaziz University, Jeddah, Saudi Arabia; ^2^ Department of Biological Science, Faculty of Science, King AbdulAziz University, Jeddah, Saudi Arabia; ^3^ Department of Medical Laboratory Technology, Faculty of Applied Medical Sciences, King Abdulaziz University, Jeddah, Saudi Arabia; ^4^ Department of Laboratory Medicine, Faculty of Applied Medical Sciences, Umm Al-Qura University, Makkah, Saudi Arabia; ^5^ College of Applied Medical Sciences, Taibah University, Madinah, Saudi Arabia; ^6^ Strategic Research and Innovation Laboratories, Taibah University, Madinah, Saudi Arabia; ^7^ Department of Medical Laboratory Technology, Faculty of Applied Medical Sciences, University of Tabuk, Tabuk, Saudi Arabia; ^8^ Department of Medical Microbiology and Parasitology, Faculty of Medicine, King Abdulaziz University, Jeddah, Saudi Arabia

**Keywords:** oncolytic virus, VSVΔ51M, interleukin-12, cancer immunotherapy, B16F10, MCF-7

## Abstract

The use of oncolytic viruses (OVs) in combination with cytokines, such as IL-12, is a promising approach for cancer treatment that addresses the limitations of current standard treatments and traditional cancer immunotherapies. IL-12, a proinflammatory cytokine, triggers intracellular signaling pathways that lead to increased apoptosis of tumor cells and enhanced antitumor activity of immune cells via IFN-γ induction, making this cytokine a promising candidate for cancer therapy. Targeted expression of IL-12 within tumors has been shown to play a crucial role in tumor eradication. The recent development of oncolytic viruses enables targeted delivery and expression of IL-12 at the tumor site, thereby addressing the systemic toxicities associated with traditional cancer therapy. In this study, we constructed an oncolytic virus, VSVΔ51M, based on the commercially available VSV wild-type backbone and further modified it to express human IL-12. Our preclinical data confirmed the safety and limited toxicity of the modified virus, VSV-Δ51M-hIL-12, supporting its potential use for clinical development.

## Introduction

The current standard of care treatments for cancer involves surgical removal of primary tumors with radiation therapy and chemotherapy as adjuvants (Łukasiewicz et al., 2021). Immune escape by tumors is a complication that has historically made cancer treatment tricky. For this reason, traditional treatment methods, such as radiation and chemotherapy, are ineffective as they do not address the systemic immunosuppression that eventually leads to relapse due to metastatic lesions (Schuster et al., 2006). Therefore, cancer immunotherapy has gained much traction due to its mechanism of revitalizing the immune system to function against cancer. The development of cancer immunotherapy resulted in immunotherapy being considered the “Breakthrough of the Year” by Science (Couzin-Frankel, 2013). These novel therapies include adaptive cell therapy (ACT), cytokines, monoclonal antibodies, checkpoint inhibitors, and oncolytic viruses (OVs) (Abbott and Ustoyev, 2019). Although cytokine therapy has potential, trials have shown that sole and systemic use of cytokines is ineffective. Nonetheless, the potent antitumor activity of cytokines still makes them indispensable; therefore, the combination of cytokines and OVs is a candidate treatment that is predicted to overcome current limitations (Waldmann, 2018). More recently, OVs have emerged as a promising option; these viruses are genetically modified to target and destroy cancer cells specifically. This has produced positive results in experimental metastasis and clinical development models, which have led to the FDA approval of oncolytic virotherapy for melanoma (Barber, 2005; Reach, 2015; Ma et al., 2016). These findings suggest that further exploration of strategies that enhance the body’s immune response to cancerous cells is necessary to treat all types of cancer. OVs provide a distinct advantage over conventional treatments because they target cancer cells specifically while leaving normal cells unharmed (Abd-Aziz and Poh, 2021). The specificity of OVs is achieved through genetic modification, including tumor-specific antigen recognition through gene editing and enhanced immune activity through the insertion of proinflammatory cytokines into the viral genome, such as IL-12.

IL-12 is a pro-inflammatory cytokine primarily produced by antigen-presenting cells, such as monocytes/macrophages and dendritic cells (Ma et al., 2015). It consists of two subunits, p40 and p35, which bind to the IL-12 receptors, IL-12Rβ1 and IL-12β2 found on cytotoxic T cells (CD8^+^ T cells) and natural killer (NK) cells (Ma et al., 2015; Mirlekar and Pylayeva-Gupta, 2021). This interaction triggers intracellular signaling pathways, including the JAK2 and TYK2 pathways, which activate STAT4, resulting in increased transcription of interferon-gamma (IFN-γ), making IL-12 a potent antitumor molecule (Guo et al., 2019). IFN-γ has been shown to induce apoptosis in tumor cells while upregulating the immune checkpoint molecules that may contribute to tumor progression (Gocher et al., 2022). Additionally, the activation of STAT4 by IL-12 leads to T-bet transcription in T cells, promoting the differentiation of T cells into helper T cells (CD4^+^ T cells), increasing the recruitment of CD8^+^ T cells and NK cells, and enhancing their antitumor activity (Mirlekar and Pylayeva-Gupta, 2021). Targeted expression of IL-12 within the tumor has been shown to play a decisive role in tumor eradication, which is supported by evidence from several preclinical studies (Alkayyal et al., 2017; Ge et al., 2020; Ghouse et al., 2020; Nakao et al., 2020) and clinical trials; for example, a phase 1 trial in high-grade human glioma revealed increased intratumoral immune cell infiltration after induction of hIL-12 using a transcriptional activator (Chiocca et al., 2019). Due to these benefits, IL-12 remains a promising candidate for cancer therapy, especially with the recent development of oncolytic viruses, which addresses the systemic toxicities associated with cancer therapy and enables targeted delivery and expression of IL-12 directly at the tumor site.

Vesiculovirus is a genus within the Rhabdoviridae family, which includes approximately 18 different genera of viruses (Walker et al., 2018). Although Vesiculovirus species can infect mammals and be transmitted by insects (Sun et al., 2010), other family members are specific to certain species. For example, Lyssavirus is capable of causing rabies only in mammalian species, while Novirhabdovirus infects only fish (Walker et al., 2018). Despite these differences, all Rhabdoviruses have some common features, including a bullet-shaped virion, a helical nucleocapsid, and a simple genome of approximately 11–16 kb that encodes five structural proteins (Walker et al., 2018). The Vesiculovirus genome has a single-stranded, negative-sense RNA structure and is 11 kb in size. It encodes five structural proteins arranged in the following order: N (nucleocapsid), P (phosphoprotein), M (matrix), G (glycoprotein), and L (large) (Redondo et al., 2015). One of the key characteristics of vesicular stomatitis virus (VSV) that makes it a promising candidate for oncolytic virotherapy is its sensitivity to interferon (IFN) signaling. This was discovered in the 1990s when it was observed that mice lacking IFN receptors were significantly more susceptible to viral infections than wild-type mice, highlighting the importance of IFN signaling in fighting viral infections (Müller et al., 1994). Cancer cells are deficient in IFN signaling, so they are particularly susceptible to oncolytic viruses such as VSV, which can specifically target and kill cancer cells while leaving healthy cells unharmed (Simovic et al., 2015). However, there are limitations to using VSV as an oncolytic virus, largely due to the toxicity of the M protein. Nevertheless, genetic manipulation of the M protein holds great promise for improving the safety and efficacy of oncolytic virotherapy. For instance, deletion of the methionine residue at position 51 in the M protein has been shown to reduce cytotoxicity and improve the safety of VSV (Kopecky et al., 2001; Hastie et al., 2013). Accordingly, we aimed to construct a VSVΔ51M virus platform and further modify it by inserting hIL-12 for potential clinical development.

## Materials and methods

### Cell lines and media

Human breast cancer cell line Michigan Cancer Foundation-7 (MCF-7) (ATCC, HTB-22), human lung carcinoma epithelial cells (A549), murine melanoma cell line (B16F10), cellosaurus GM00038 9Y fibroblast (GM-38) (CVCL-7271), baby hamster kidney cell line (BHK-21/WI-2) (Kerafast, EH1011), and African green monkey kidney-derived Vero-E6 cell line (ATCC, CRL-1586) were cultured in Dulbecco’s modified Eagle medium (DMEM-1X) (Thermo Fisher, Waltham, MA) containing penicillin (100 U/ml) and streptomycin (100 μg/ml) and supplemented with 5 or 10% (v/v) heat-inactivated fetal bovine serum (FBS) (Biosera Europe, France) in a 5% CO_2_ environment at 37°C. Cell lines were passaged every 3 days and maintained in culture for no more than 2 weeks before experiments began.

### Construction of recombinant VSV vectors

To generate a recombinant VSV (rVSV) plasmid, the glycoprotein gene of VSV *(VSV-G)* was amplified from a pCAGGS-G-Kan plasmid EH1017 (Kerafast, Boston, MA) and subsequently inserted between *MluI* and *NheI* restriction sites of the VSVΔG-P/M-MCS2-2.6 EH1033 expression plasmid (Kerafast, Boston, MA) at its original position between matrix *M)* and polymerase *L)* genes using the following primers: forward 5′-GAT​CAC​GCG​TAT​GAA​GTG​CC-3′ and Reverse 5′-GAT​CGC​TAG​CTT​ACT​TTC​CAA​GTC​G-3’. A single methionine deletion at position 51 of the matrix *M)* protein (MΔ51) in VSV plasmid was then performed by inverse PCR using the primers designed to be compatible with the In-Fusion^®^ HD Cloning Kit (Takara Bio Inc., San Jose, CA) as the following: forward 5′-TTG​ACG​AGG​ACA​CCT​ATG​ATC​CGA​ATC​AAT​T3′ and reverse 5′-AGG​TGT​CCT​CGT​CAA​CTC​CAA​AAT​AGG​ATT-3’. The generated VSVΔ51M construct was then used as a parental vector to generate VSVΔ51M-hIL-12. The *hIL-12* was amplified from puno1-hil12ba plasmid (InvivoGen, France) by PCR to be incorporated between *AscI* and *AvrII* restriction sites using the following primers: forward 5′-GTA​TGG​CGC​GCC​GCC​ACC​ATG​TGT​CAC​CAG​CAG​TTG​GTC​A-3′ and reverse 5′-TAC​CCC​TAG​GTC​ATA​GGA​AGC​ATT​CAG​ATA​GCT​C-3′ and subsequently ligated to the VSVΔ51M plasmid by T4 DNA ligase (New England Biolabs, Ipswich, MA). Construction of generated plasmids was confirmed by sequencing and restriction digestion.

### Rescue of recombinant VSV viruses

To rescue the recombinant VSV, VSVΔ51M, and VSVΔ51M-hIL-12, 70% confluent BHK-21 cells were infected with a recombinant vaccinia virus expressing T7 RNA polymerase that is required to initiate transcription at a multiplicity of infection (MOI) of 10. After 1h 30 min incubation, cells were transfected with 2 μ g DNA plasmid harboring the viral genome, in addition to VSV N, P, G, and L helper plasmids (1, 1.25, 1.25, and 0.25 μ g, respectively) using Lipofectamine 2000 Transfection Reagent (Thermo Fisher, Waltham, MA) (Cat: 11668019) according to the manufacturer’s instructions. After 48 h post-transfection, the rescued virus was collected, filtered by 0.22 µm filter to remove the remaining vaccinia, and passaged on Vero-E6 cells until apparent cytopathic effects (CPE) were observed.

### Viral plaque assay

The rescued viruses were serially diluted in serum-free DMEM (10-fold dilutions), and the viral suspension was used to infect Vero-E6 monolayer 500 μL/well in a six-well plate in duplicate. After 1 h incubation, the virus inoculum was removed, and a mixture of 1.6% Bacto-Agar-Dehydrated (Thermo Fisher, Waltham, MA) and 2X DMEM (Thermo Fisher, Waltham, MA) overlay containing 20% FBS at the 1:1 ratio was added (2 ml/well). Plaques were fixed following 24 h incubation at 37°C after infection with methanol and acetic acid mixture (3:1) and stained with Coomassie blue to visualize the plaques. The titer of viral stocks was quantified and calculated in plaque-forming units (PFU/ml) using the following formula Viral titer = (number of viral plaques x 2) x dilution factor.

### Reverse transcription-polymerase chain reaction (RT-PCR)

Gene expression analysis was performed to validate the successful recovery of each virus utilizing RT-PCR. Vero-E6 cells were seeded into a 6-well plate to reach 90% confluency. After 24 h of incubation, the cells were either mock infected or infected with VSV, VSV-Δ51M or VSV-Δ51M-hIL-12 in DMEM without FBS at an MOI of 10. The viral suspension was then aspirated after 1 h of adsorption period and replaced with a fresh DMEM media containing 5% FBS. Cells were harvested 12 h post-infection, and the total RNA was extracted using a RNeasy kit per manufacturer’s instructions (Qiagen, Germany) (Cat: 74106). RNA expression of VSV genes (N, P, M, and G) and hIL-12 from rescued recombinant viruses was confirmed using a high-capacity Superscript III Platinum™ One-Step qRT-PCR Kit (Thermo Fisher, Waltham, MA) (Cat: 11732088) according to the manufacturer’s instructions.

### Viral kinetics assays

Vero-E6 cells were seeded into a 24-well plate until reaching 90–95% confluence after 24 h of incubation. VSV, VSVΔ51M, or VSVΔ51M-hIL-12 viruses were used to infect the cells in FBS-free media at 10 MOI for a single step or at 0.01 MOI for multi-step growth kinetics. Post-infection, each virus supernatant was collected at different time points 6, 12, 18, 24, and 48 h and stored at -80°C for plaque assay titration (each virus infection was conducted in 3 biological replicates for each time point). The yield of each virus at each time point was expressed as PFU/ml and determined by plaque assay as described above.

### Indirect immunofluorescence

Approximately 90% confluent Vero-E6 cells in a cell culture slide were infected with VSV, VSVΔ51M or VSVΔ51M-hIL-12 virus at 0.1 MOI. After 18 h incubation at 37°C in a 5% CO_2_ incubator, the media was discarded, and attached cells were washed with PBS. Then, 4% formaldehyde was used for fixation at 4°C for 10 min. After fixation, cells were permeabilized with 0.2% PBS-Triton X-100 (PBS-Triton) at 4°C for 20 min, washed twice with PBS-Triton, and blocked with PBS-Triton containing 2% goat serum for 30 min at room temperature. Cells were then washed with PBS-Triton and stained with primary antibodies. The following primary antibodies; mouse anti-VSV-G (clone 8G5F11), mouse anti-VSV-M (clone 23H12), and mouse anti-VSV-N (clone 10G4) (Kerafast, Boston, MA) were used at a dilution of 1:1000 in blocking buffer and incubated at 4°C for 18 h. Cells were then washed three times, and strained with goat anti-mouse IgG secondary antibody labeled with Alexa Flour-555 (Abcam, United Kingdom) diluted in blocking buffer at a dilution of 1:500 and incubated in the dark at room temperature for 1 h. Cells were then washed with PBS-Triton three times, and then VECTASHIELD with DAPI counterstain antifade mounting medium was mounted to the slide for visualization (Vector Laboratories, Inc., Burlingame, CA). Fluorescence Microscope Olympus BX51 was used to capture the images at 40X, and ImageJ 1.53e Software (Rueden et al., 2017) was used for the analysis.

### Western blot

Cultured Vero-E6 cells infected with VSV, VSVΔ51M or VSVΔ51M-hIL-12 at an MOI of 0.1 were harvested after 24 h of infection and lysed by RIPA lysis buffer (Sigma, Germany). Then, 25 µg of total protein were loaded onto 8% SDS-PAGE and transferred to a polyvinylidene difluoride (PVDF) membrane to detect the expression of G protein. The membrane was blocked with 5% skimmed milk in Tris-buffer saline (TBS) and 0.05% Tween-20 (TBS-T) for 1 h at room temperature, and incubated at 4°C with the primary monoclonal mouse anti-VSV-G (clone 8G5F11) antibody (1:1000 dilution) in TBS-T mixed with anti-alpha-tubulin antibody (Rockland Immunochemicals, Pottstown, PA) at 1:2000 dilution. The membranes were then washed on the following day with TBS-T and incubated with horseradish peroxidase conjugated secondary anti-mouse IgG antibody (1:2000) (Sigma, Germany) for 1 h at room temperature. The blot was scanned, and protein density was measured using iBright FL1500 (Thermo Fisher, Waltham, MA).

### Measurement of secreted hIL-12 cytokine

MCF-7 and Vero-E6 were seeded into 24-well plates (1 × 10^5^ cells/well) and incubated overnight to reach 90% confluency. The next day, cells were infected with VSVΔ51M or VSVΔ51M-hIL-12 at MOI of 10 in 2 biological replicates. Supernatants were collected from cells at different time points (18, 24, and 48 h) post-infection and assayed for hIL-12 cytokine expression using human IL-12 (p70) ELISA Kit (Thermo Fisher, Waltham, MA) according to the manufacturer’s guidelines.

### Transmission electron microscopy

Briefly, confluent Vero-E6 cells were infected with the VSVΔ51M virus. After overnight incubation, the supernatant was collected in spl 50 ml conical tube (Cat: 50050) and purified by low-speed centrifugation (350 g, 10 min) at room temperature to remove cell debris and subjected to sucrose cushion high-speed ultracentrifugation (188,000 g, 1.5 h) at 4°C. Then, the supernatant was collected, and the virus pellet was resuspended in 1 ml PBS, and 50 µL of the virus were fixed by 4% glutaraldehyde for transmission electron microscope image analysis at the Imaging and Characterization Core Lab in the core facility at KAUST Thuwal, Saudi Arabia (https://corelabs.kaust.edu.sa/labs/imaging-and-characterization-core-lab).

### Microscopic analysis of infected cancer cells

Cancer cell lines including MCF-7, A549, GM-38, and B16F10 cells were cultured at approximately 90% confluency in 6-well plates and mock-infected or infected with VSV, VSVΔ51M or VSVΔ51M-hIL-12 at MOI of 0.1. Representative images were captured with an EVOS M7000 microscope (Thermo Fisher, Waltham, MA) at 24 h post-infection.

### Viability assay

Cancer cell lines including MCF-7, A549, B16F10, and GM-38 fibroblasts were cultured into 96-well plates (2 × 10^4^ cells/well). After 24 h of incubation; cells were infected with VSV, VSVΔ51M or VSVΔ51M-hIL-12 viruses at different MOIs (10, 1, 0.1, 0.01, and 0.001 PFU/cell), and incubated at various time points (48, 72, and 96 h). At each time point, the supernatant was removed and replaced with 90 μL/well fresh serum-free media and 10 μL/well Alamarblue™ HS Cell Viability Reagent (Thermo Fisher, Waltham, MA) (Cat: A50100) and incubated at 37°C, 5%. CO_2_. Absorbance was measured at 570 nm after 4 h using BioTek Synergy 2 microplate reader (BioTek, Winooski, VT).

### Viral infection of MCF-7 cells and co-culture with PBMCs

Confluent MCF-7 cells were mock infected or infected with VSVΔ51M or VSVΔ51M-hIL-12 (MOI = 0.1). After 24 h of infection, cells were harvested and co-cultured with isolated peripheral blood mononuclear cells (PBMCs) from a healthy donor at (1:2) ratio in RPMI medium 1640 (Thermo Fisher, Waltham, MA) (Cat: 11875093) in U-bottom 96-well plates. For flow cytometry analysis, co-cultured cells were treated with Protein Transport Inhibitor Cocktail (BD Biosciences, San Jose, CA) at a dilution of 1:1000 for 4 h at 37°C and 5% CO_2_. PBMCs were stimulated with phorbol 12-myristate 13-acetate (PMA) and ionomycin I) as positive control, and in fresh RPMI 1640 medium as negative control. After stimulation, cells and supernatants were used for cytokine production using FACS and ELISA analysis. All experiments on human samples were conducted upon ethical approval from the Research Ethics Committee (REC) in the Unit of Biomedical Ethics King Abdulaziz University Hospital (KAUH), King Abdulaziz University (KAU), Jeddah, Saudi Arabia (Reference No 193–22).

### Flow cytometry analysis

The expression of IFN-γ response from stimulated NK cells was evaluated by flow cytometry after 4 h of co-culture. In brief, cells were washed with PBS twice and stained with a LIVE/DEAD™ Fixable viability stain 780 (BD Biosciences, San Jose, CA) (Cat: 565388) for 15 min at 4°C. After washing with FACS buffer (PBS with 2% heat inactivated FBS), PE-conjugated anti-human CD56 and FITC-conjugated anti-human CD3 and (BioLegend, United Kingdom) were used for extracellular staining. Cells were then washed with FACS buffer, fixed, and permeabilized using Cytofix/Cytoperm Solution (BD Biosciences, San Jose, CA) (Cat: 554655) according to the manufacturer’s protocol. Similarly, cells were stained and labeled intracellularly with Alex700-conjugated anti-human IFN-γ (clone 4S.B3) for 20 min at 4°C. Cells then were washed twice with permeabilization buffer and once with FACS buffer before being processed for flow cytometric analysis using the BD FACSAria™ III flow cytometer (BD Biosciences, San Jose, CA) and FlowJo v10 software (Tree Star).

### Cytokine quantification with enzyme-linked immuno-sorbent assay (ELISA)

The production and secretion of IFN-γ from stimulated PBMCs were evaluated using ELISA MAX™ Standard set Human IFN-γ according to the manufacturer’s instructions (Biolegend, United Kingdom). Briefly, the primary antibody was incubated overnight at 4°C in a coating buffer on high protein-binding 96-well plates (EU Immulon 2 HB plates). After washing with PBS containing 0.1% Tween-20 (PBS-T) and blocking with 1% BSA in PBS for 2 h at room temperature, the cytokine standards and supernatant samples were added and incubated for 2 h at room temperature, followed by incubation for 1 h with a detection antibody. After 30 min incubation with Avidin-HRP, 100 μL of TMB substrate solution were added, and the reaction was stopped with the stop solution after 20 min. The absorbance was measured immediately at 450 nm using a Synergy 2 Multi-Detection Microplate Reader (Bio Tek, Winooski, VT). Concentrations of secreted IFN-γ in supernatants were determined from the standard curve.

### *In vivo* studies

A total of 21 female C57BL/6 mice, aged between 6 and 8 weeks, were obtained from the animal facility unit at King Fahd Medical Research Center (KFMRC), KAU, Jeddah, Saudi Arabia. Animals were housed in sterile conditions, and all experiments were conducted following the guidelines and approval of the Institutional Animal Care and Use Committee (IACUC) at KFMRC, KAU, Jeddah, Saudi Arabia (Reference No ACUC-20–03-9-10) and ethical approval from the bioethical committee at KAU (Reference No 04-CEGMR-Bioeth-2020). Subcutaneous B16F10 melanoma tumors were established by injecting cell suspensions (5 × 10^5^ cells) into the right flanks of female C57BL/6 mice. Treatment was initiated once the tumor was established (palpable or measuring ∼25 mm^2^ in size). Tumors were developed after ∼7 days of tumor initiation, after which mice were randomized according to tumor establishment and size into 3 groups, VSV, VSVΔ51M, and untreated group (7 mice/group). Following randomization, 5 × 10^8^ PFU of VSV or VSVΔ51M per dose in 50 μL of sterile PBS was administered intratumorally on days 7, 10, and 13 after tumor formation. The tumor size was measured three times a week, where tumor volume was calculated using the formula: Tumor volume mm^2^ = length × width^2^.

### Statistical analysis

All statistical analyses were generated via GraphPad Prism version 8.0 software (Graph-Pad Software, Inc., San Diego, CA). For viral titers, the logarithm was calculated before statistical analysis. All tests were analyzed by variance analysis of multigroup comparisons using One or Two-way ANOVA analysis with Tukey’s multiple post-hoc comparisons test. The survival differences between tumor-bearing and treated mice were evaluated using the Kaplan-Meier survival curve and analyzed by the Log-rank (Mantel-Cox) test. All values are illustrated as mean ± SD, and statistical significance is reported as *, *p* ≤ 0.05, **, *p* ≤ 0.01, ***, *p* ≤ 0.001 and ****, *p* ≤ 0.0001.

## Results

### Construction and rescue of VSV, VSVΔ51M, and VSVΔ51M-hIL-12 viruses

**FIGURE 1:**
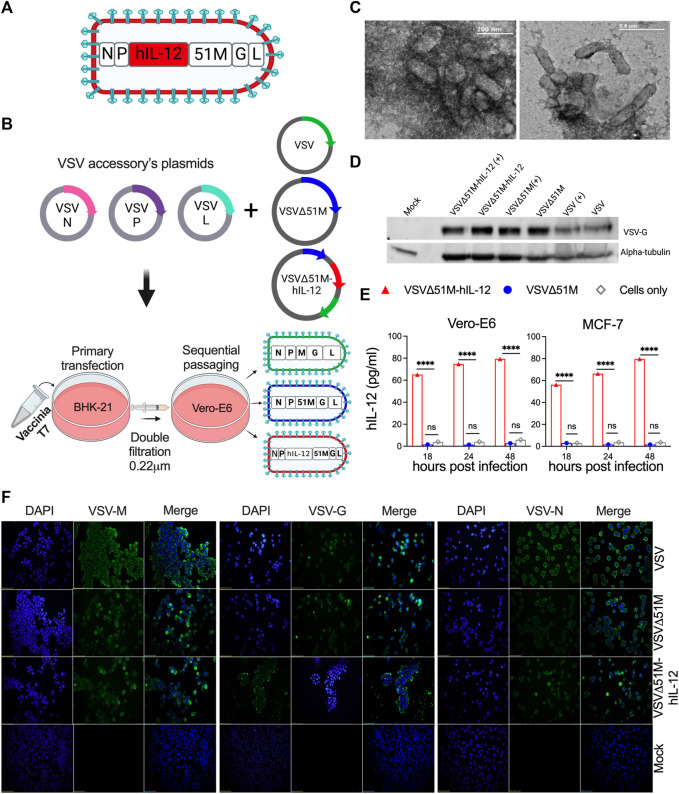
Construction and generation of VSV viruses. **(A)** Schematic diagram of the recombinant VSVΔ51M-hIL-12 virus. The *hIL-12* gene is indicated in red and was inserted between the *M and P* genes of the parental vector VSVΔ51M using the *AscI* and *AvrII* restriction sites. **(B)** Schematic representation of the generation process for the VSV, VSVΔ51M and VSVΔ51M-hIL-12 viruses. BHK-21 cells were infected with vaccinia T7; transfected with the targeted plasmid VSV, VSVΔ51M or VSVΔ51M-hIL-12 and VSV-system accessory plasmids; and then primary supernatant (Passage 1) was used for sequential passaging in Vero-E6 cells to propagate recovered viruses. **(C)** Negative staining of electron micrographs of purified VSVΔ51M virions. Fifty microliters of the purified virus were collected, fixed in 4% glutaraldehyde, and imaged using transmission electron microscopy. Arrows indicate the magnified area of the virus with a bullet-shaped morphology. **(D)** Western blot analysis of recovered VSV virus-infected cells. MCF-7 cells were infected at an MOI of 10 with VSV, VSVΔ51M or VSVΔ51M-hIL-12 or transfected with each virus control plasmid. Cell lysates were prepared, separated by 8% SDS‒PAGE, and electrophoretically transferred to a PVDF membrane. The VSV-G protein was detected with anti-VSV G antibody. **(E)** Production of hIL-12 by MCF-7 or Vero-E6 cells infected with VSVΔ51M or VSVΔ51M-hIL-12 at different time points, as measured by ELISA. The expression level of hIL-12 was detected at 18, 24, and 48 h post-infection with VSVΔ51M-hIL-12 and is reported as pg/ml of hIL-12. Mock-infected control cells and cells infected with parental VSVΔ51M failed to produce a detectable level of hIL-12. Data were analyzed using two-way ANOVA with Tukey’s multiple comparisons test, n = 3; the graph represents the mean ± SD (****p < 0.0001). **(F)** Recombinant VSV protein expression and localization. Immunofluorescence staining of Vero-E6 cells infected with VSV, VSVΔ51M or VSVΔ51M-hIL-12 or transfected with control plasmids. Infected and transfected cells were stained with anti-VSV N, M, or G mouse monoclonal antibodies (green), and nuclei were counterstained with DAPI (blue). Scale bars are included in each image.

A panel of genetically modified viruses, including VSV, VSVΔ51M, and novel VSVΔ51M-hIL-12, were constructed based on the VSV Indiana strain reverse genetics system. The G gene was incorporated between the M and L genes of VSVΔG to generate VSV. The VSVΔ51M construct was then created by the generation of an 51M deletion. Following this modification, the hIL-12 gene was inserted between the M and P genes of VSVΔ51M to generate VSVΔ51M-hIL-12 (Figures 1A,B). Prior to viral rescue, we confirmed the 51M deletion and hIL-12 gene insertion by colony PCR screening, sequencing, and restriction digestion (Supplementary Figure S1). After successful viral rescue from all constructs, we evaluated whether the rescued viruses induced CPE in cultured cells. Indeed, all viruses resulted in a typical VSV CPE, which was characterized by detached and rounded cells after 24 h of infection (Supplementary Figure S2A). To validate the transcriptional activity of rescued viruses, RT-PCR findings showed that VSV genes, including G (650 bp), M (690 bp), N (402 bp), P (814 bp), and the inserted hIL-12 gene (1536 bp) indicated efficient expression in the engineered viruses compared to their corresponding positive control viruses, as shown in Supplementary Figure S2B.

Furthermore, the immunocytochemical staining analysis showed that the M, G, and N proteins were expressed and localized within the appropriate primary intracellular compartments in infected cells but not mock-infected cells (Figure 1F). The glycoprotein of each virus was detected by Western blotting, which showed bands corresponding to the expected size of the VSV-G protein (∼63 kDa) (Figure 1D). Having demonstrated that all viral genes were expressed, we wanted to assess whether the generated VSV viruses retained their bullet-shaped virions. As illustrated in Figure 1C, electron microscopy imaging showed that the VSVΔ51M virus has a typical bullet shape of Rhabdoviruses. Finally, to confirm the expression of hIL-12 by the VSVΔ51M-hIL-12 virus, we measured hIL-12 secretion upon infection of MCF-7 or Vero-E6 cells with VSVΔ51M or VSVΔ51M-hIL-12 at an MOI of 10 at different time points (18, 24, and 48 h). Indeed, hIL-12 was significantly expressed at high levels in MCF-7 and Vero-E6 cells infected with VSVΔ51M-hIL-12 but not in the control groups, including parental VSVΔ51M - or mock-infected cells, across all different time points (*p* < 0.0001) (Figure 1E). All generated viruses were amplified, purified and titrated by plaque assay and representative plaques are shown in Supplementary Figure S3. Taken together, these findings demonstrated that all viruses were successfully rescued and that their cytotoxic functions were retained.

### Recombinant VSVΔ51M virus retains its therapeutic efficacy in a B16F10 subcutaneous tumor model

**FIGURE 2:**
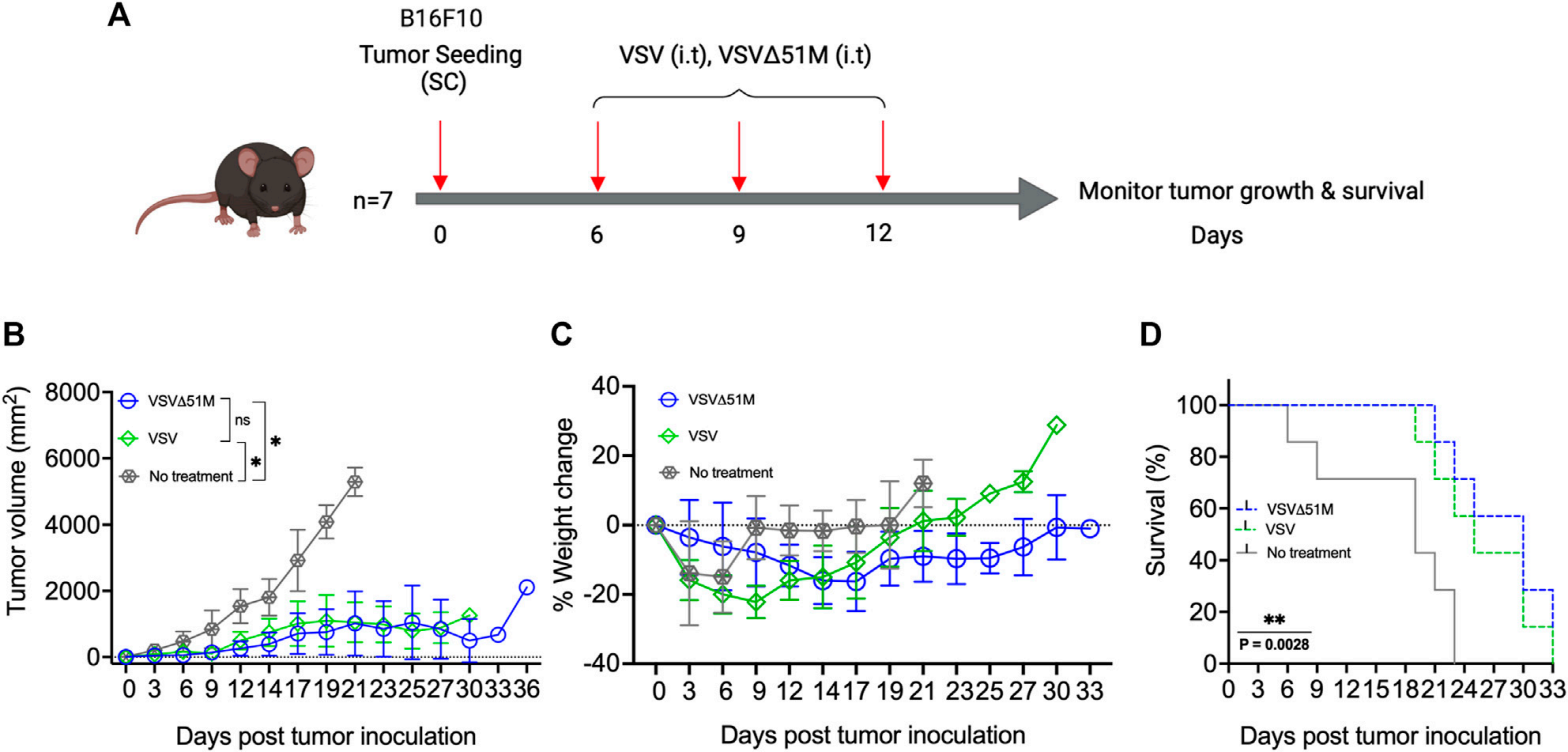
Efficacy of VSV and VSVΔ51M in C57Bl/6 mice bearing B16F10 tumors. **(A)** Timeline of animal experiment. Six-to eight-week-old female C57Bl/6 mice were subcutaneously injected with 100 μL of 5 × 10^5^ B16F10 cells in the right flank on Day 0. Tumors were established by day 7, and mice were randomly divided into 3 groups (n = 7 mice per group). One group served as a control (no treatment), and three intratumoral doses of VSV or VSVΔ51M (5 × 10^8^ PFU/50 μL), starting on day 7 (days 7, 10, and 13), were administered to the other two groups. **(B)** Tumor size was monitored in each group by caliper measurements until a mouse in the group was euthanized due to its tumor burden. Tumor size was calculated using the following formula: tumor volume (mm^2^) = length × width^2^. **(C)** Mice were weighed every 3 days, and the percentage of weight change of each mouse was determined. **(D)** The survival times of treated and untreated groups were plotted as a Kaplan‒Meier survival curve. Statistical analysis of tumor volume was performed by two-way ANOVA, followed by survival analysis by the log-rank (Mantel‒Cox) test (**p = 0.0028). *p < 0.03; **p < 0.002; ***p < 0.0002; ****p < 0.0001.

To investigate the potential therapeutic benefits of VSVΔ51M as an oncolytic agent, 8-week-old C57Bl/6 mice were subcutaneously inoculated with 5 × 10^5^ B16F10 melanoma cells (100 µL). The tumors were palpable ∼7 days after implantation. The mice were then treated with three doses of either VSV or VSVΔ51M at 5 × 10^8^ PFU/50 µL on days 7, 10, and 12 (Figure 2A). The tumor outgrowth of mice treated with VSV or VSVΔ51M showed a significant delay in progression compared to that of untreated mice (*p* = 0.01) (Figure 2B). As expected, the delay in tumor outgrowth was reflected in the percentage weight change, as well as the significantly prolonged survival of both VSV and VSVΔ51M treated mice compared to untreated mice, with median survival times of 25 days, 30 days, and 19 days post tumor seeding, respectively (*p* = 0.002) (Figures 2C,D).

### Replication and growth kinetics of the generated VSV viruses

**FIGURE 3:**
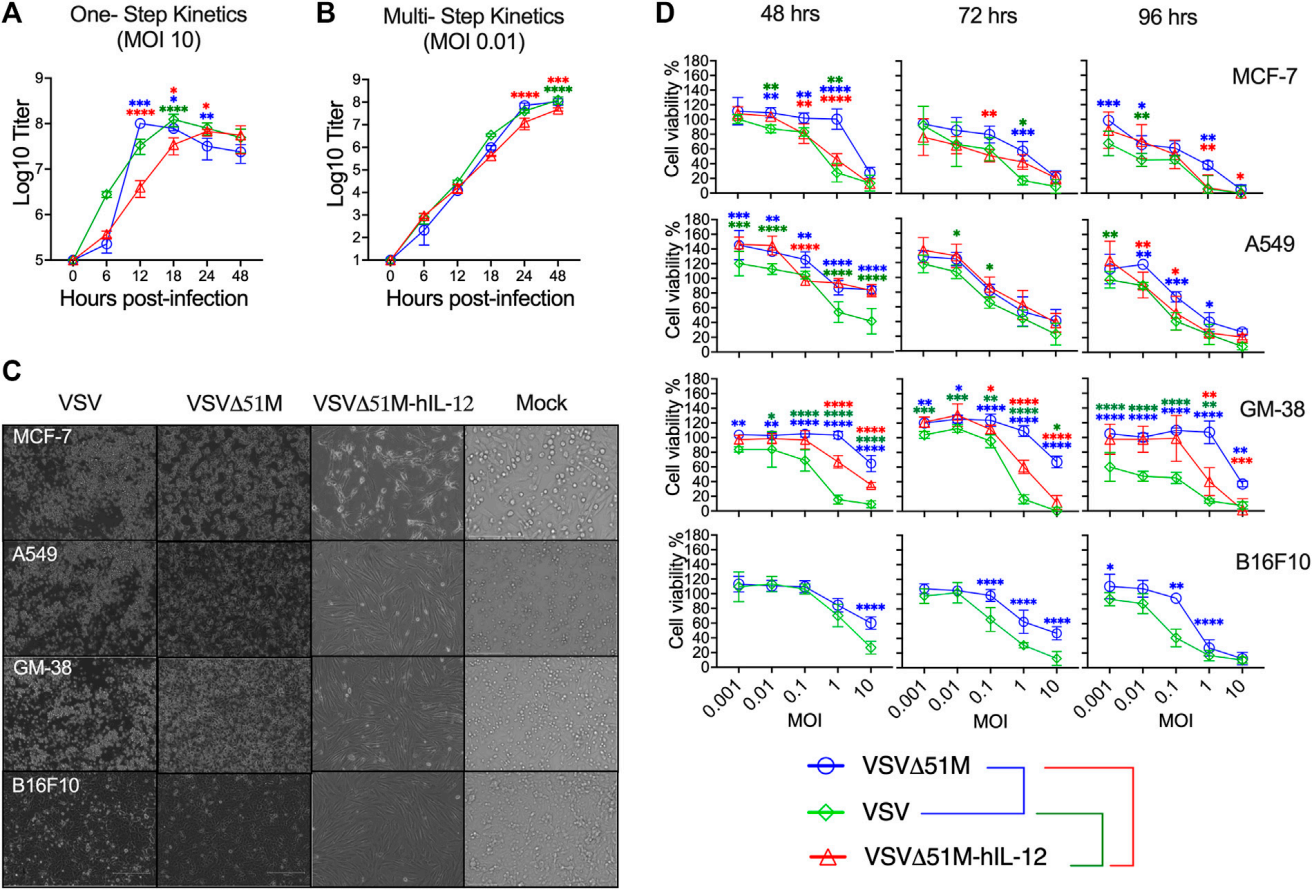
Growth and spreading kinetics and cytotoxicity of the rescued VSVs. To assess **(A)** single-step and **(B)** multistep viral growth kinetics, Vero-E6 cell monolayers were infected in triplicate with VSV, VSVΔ51M, or VSVΔ51M-hIL-12 at a multiplicity of infection of 10 or 0.01. Supernatants were collected at 6, 12, 18, 24, and 48 h after infection. The titer of each virus was determined in Vero-E6 cells (in triplicate) using standard plaque assay. The mean ± SD of log-transformed titers is shown. Statistical significance was determined using two-way ANOVA with Tukey’s multiple comparison test. **(C)** Effects of VSV viruses on the morphological characteristics of normal and cancerous cells. Light microscopy images of MCF-7, A549, GM-38, and B16F10 cells that were mock-infected or infected with VSV, VSVΔ51M, or VSVΔ51M-hIL-12 (MOI = 10) for 24 h. The cytotoxic effects of the viruses were assessed by examining representative photomicrographs of cell monolayers at a magnification of ×20 following infection. **(D)** Cytotoxicity of the VSV, VSVΔ51M, and VSVΔ51M-hIL-12 viruses to MCF-7, A549, GM-38, and B16F10 cells. Cells were infected with the VSV viruses at multiplicities of 10, 1, 0.1, 0.01, and 0.001 PFU per cell. At the indicated time points post-infection, cell viability was measured by an AlamarBlue assay. Data are expressed as the percentage of the cell viability of mock-infected cells and represent the means ± SD of 6 technical replicates. Statistical significance was determined by two-way ANOVA with Tukey’s multiple comparisons test (*p < 0.05; **p < 0.01; ***p < 0.001; ****p < 0.0001). The statistical significance calculations for the multiple comparisons have been color-coded by lines as illustrated in the figure legend underneath panel **(D)**.

Next, we sought to assess the replication and spreading kinetics of the generated VSV viruses. Vero-E6 monolayers were infected with VSV, VSVΔ51M, or VSVΔ51M-hIL-12 at an MOI of 10 or 0.01. Following infection, supernatants were harvested at multiple time points and tittered on Vero-E6 cells to measure the production of infectious progeny with the standard plaque assay method. In a one-step growth curve, the replication of VSVΔ51M reached the highest titer of 1 × 10^8^ PFU/ml and plateaued at 12 h post-infection, whereas VSV reached the same titer of 1 × 10^8^ PFU/ml at 18 h before reaching a plateau. Both viruses yielded significantly higher viral titers than VSVΔ51M-hIL-12, which plateaued at 24 h post-infection, with a titer of 6.8 × 10^7^ PFU/ml (Figure 3A). Additionally, a multistep growth curve (Figure 3B) showed that the replication rates in VSVΔ51M and VSV infected cells reached as high as 1.07 × 10^8^ PFU/ml and 1.24 × 10^8^, respectively, after 48 h, whereas VSVΔ51M-hIL-12 infected cells had significantly lower production (4.8 × 10^7^) than VSVΔ51M or VSV infected cells. These results suggested that the insertion of hIL-12 attenuated viral replication to a certain extent due to the insertion location of hIL-12, which was upstream of the M protein and, therefore, closer to the viral promoter, leading to a decrease in subsequent expression (Finke and Conzelmann, 2005). Nonetheless, it is important to note that an MOI of 10, which was used for the one-step growth kinetics analysis, is never achievable during oncolytic virus treatment *in vivo* (Murphy et al., 2012).

Furthermore, we assessed whether the oncolytic properties of the rescued viruses were retained in tumor cells. To this end, a tumor cell panel consisting of MCF-7, A549, B16F10 and GM-38 cells were infected with VSV, VSVΔ51M, or VSVΔ51M-hIL-12 and assayed with an AlamarBlue cytotoxicity assay 48, 72 and 96 h later. As shown in Figures 3C,D, MCF-7 and A549 cancer cells infected with the VSV, VSVΔ51M, or VSVΔ51M-hIL-12 viruses showed significant reductions in viable cells with more prolonged incubation and higher virus concentration. In addition, MCF-7 cells also showed increased susceptibility to VSVΔ51M-hIL-12 infection-mediated cell killing than parental VSVΔ51M. This is mostly due to the toxicity of hIL-12 carrying oncolytic virus when compared to non-cytokine carrying 51M mutation (VSVΔ51M). However, normal GM-38 cells infected with VSVΔ51M or VSVΔ51M-hIL-12 showed less cytotoxicity and retained viability compared with rVSV virus infections at MOIs less than 10. Further comparable results were also observed on murien B16F10 infected cells with VSV or VSVΔ51M at different MOIs. A significant toxic effect of VSV was observed at high MOI (MOI = 10) after 48 h of infection compared to VSVΔ51M infection. This effect was further detected at lower concentrations and more prolonged incubation. Based on this finding, infection with a high MOI and a more extended incubation period are required to increase the effectiveness of VSV replication. As a consequence of the above findings, VSV was able to infect and kill a wide range of cancer cell types, and the VSVΔ51M or VSVΔ51M-hIL-12 viruses were as effective at killing these tumor cell lines as VSV.

### VSVΔ51M-hIL-12 enhances human IFN-γ secretion *ex vivo*

**FIGURE 4:**
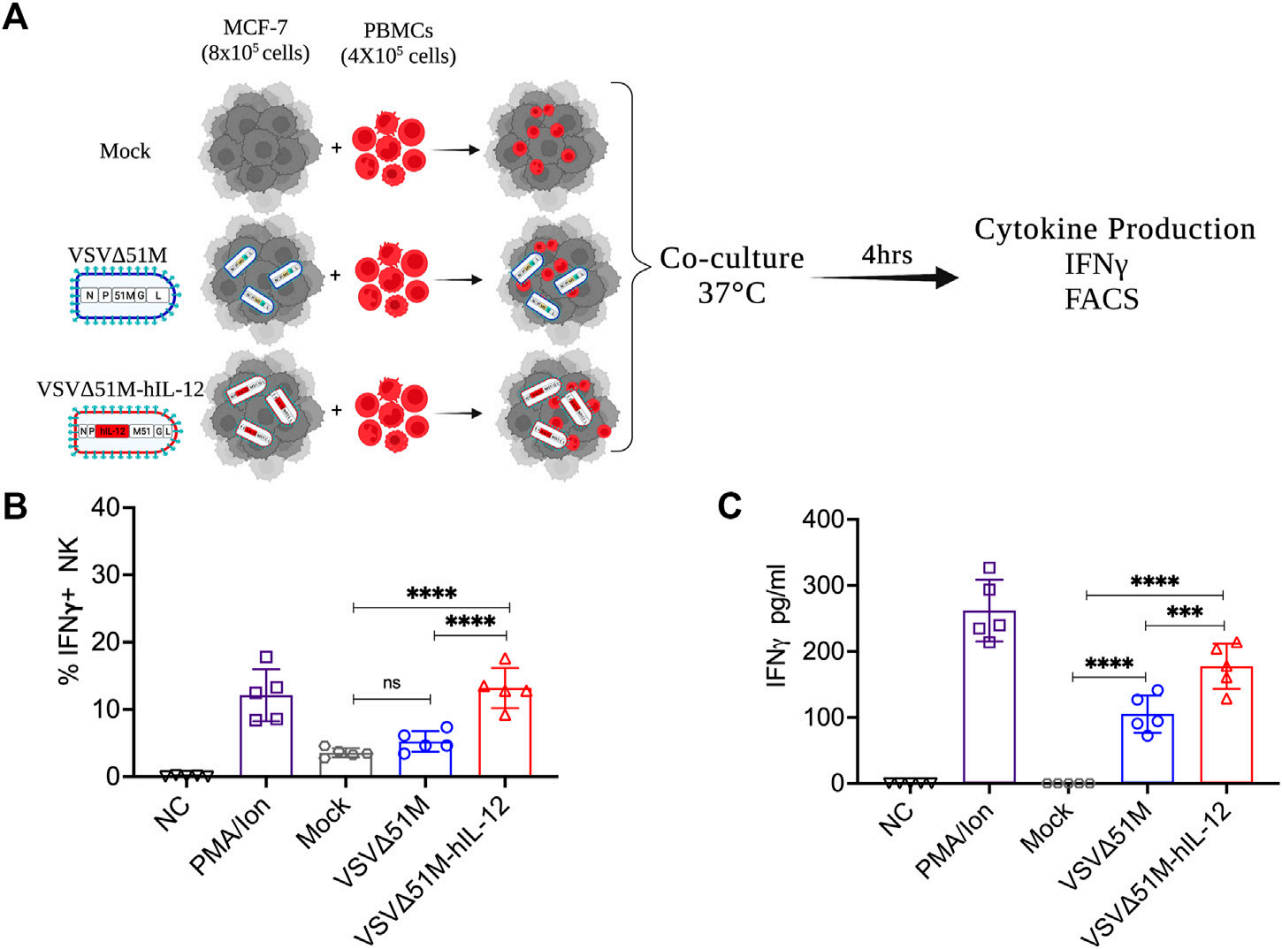
VSVΔ51M-hIL-12 enhances the production of IFN-γ by stimulated PBMCs *ex vivo.* **(A)** Schematic illustration of the experimental setup. Isolated PBMCs were cocultured with mock-infected MCF-7 cells or MCF-7 cells infected with VSVΔ51M or VSVΔ51M-hIL-12. **(B)** The percentage of intracellular IFN-γ^+^ expression by NK (CD56^+^CD3^−^) cells for each condition was quantified by flow cytometry. **(C)** The concentration of secreted IFN-γ in supernatant for each condition was quantified by ELISA. Graph represents the mean ± SD of five replicate values from two independent experiments. Differences between treatment arms were analyzed using Tukey’s multiple comparisons test (*p < 0.05; **p < 0.01; ***p < 0.001; ****p < 0.0001).

Having observed the oncolytic activity of VSVΔ51M-hIL-12, we aimed to verify that hIL-12 secretion by VSVΔ51M-hIL-12 could in fact activate human NK and other immune cells. Thus, we infected MCF-7 breast cancer cells with either VSVΔ51M or VSVΔ51M-hIL-12 and then co-cultured these infected cells with normal PBMCs obtained from a healthy donor (Figure 4A) to measure IFN-γ expression and secretion by NK and other immune cells. As shown in Figure 4B, while mock- and VSVΔ51M -infected cells resulted in insignificant intracellular expression of IFN-γ by CD56^+^CD3^−^ NK cells, co-culture of VSVΔ51M-hIL-12 -infected MCF-7 cells with normal PBMCs resulted in significantly high levels of intracellular IFN-γ in CD56^+^CD3^−^ NK cells to levels similar to those observed in PBMCs stimulated with potent mitogens such as PMA and ionomycin. Next, we measured the levels of secreted IFN-γ from the immune cells in *ex vivo* stimulated PBMCs. Secretion of IFN-γ was found to be significantly higher in supernatant obtained from PBMCs co-cultured with MCF-7 cells infected with VSVΔ51M-hIL-12 compared to those co-cultured with mock- or VSVΔ51M -infected MCF-7 cells (Figure 4C). These findings indicate that VSVΔ51M-hIL-12 secrets functional hIL-12 from infected cells capable of activating CD56^+^CD3^−^ NK cells and other immune cells in PBMCs to express and secrete IFN-γ. Our results support the hypothesis that VSVΔ51M-hIL-12 could produce a much-needed therapeutic benefit in treating cancer.

## Discussion

A new class of cancer treatments is represented by tumor-targeted replication-competent viruses (Shin et al., 2007). Therefore, using OVs to treat tumors on multiple fronts is emerging as a promising clinical treatment option. The most crucial feature of these viruses is that they stimulate antitumor immune responses when propagated in permissive tumors (Lemay et al., 2012). In addition, developing oncolytic agents for cancer treatment based on viruses with tumor-specific properties has become increasingly imperative. Accordingly, VSV has several favorable properties that make it an attractive OV candidate for clinical trials, including its small, easily manipulable genome, rapid replication kinetics, absence of VSV-specific neutralizing antibodies in humans, and broad oncotropism (Simovic et al., 2015; Felt and Grdzelishvili, 2017). In addition, as type I interferons (IFNs) protect normal cells from VSV infection-mediated lysis, VSV cannot cause transformation in healthy cells. However, it has been demonstrated that wild-type VSV exhibits cytotoxic properties due to its M protein inhibiting both cellular transcription and nucleocytoplasmic mRNA transport (Hoffmann et al., 2010). Therefore, to avoid VSV-induced cytotoxicity, molecular engineering of VSV through deletion or mutation of virulence genes has been proven safe by replicating exclusively in cancer cells. This unique characteristic expands the utility of VSV in settings other than cancer immunotherapy, such as an antiviral vaccine platform. Based on these facts, in this study, we aimed to develop our own strain of VSVΔ51M from a commercially available glycoprotein-deleted VSV plasmid designed for VSV pseudotyping studies. We restored the full VSV genome by reinserting the glycoprotein and deleted the 51M residue in the matrix gene of the virus to attenuate its ability to infect healthy/normal cells while gaining its oncolytic effect, as reported previously (Stojdl et al., 2003).

In this study, we performed an extensive characterization and confirmation of the VSVΔ51M platform to support our claims in alignment with the reported literature pertaining to VSVΔ51M. For instance, we confirmed the presence of VSVΔ51M proteins by immunofluorescence and western blotting. The expression of the M, G, and N proteins in VSVΔ51M -infected cells showed that these proteins retained their structural integrity and cellular localization (Figures 1D,F). The western blotting result was similar to those obtained by *Ayala-Breton et al.*, who confirmed the expression of the VSV-G, VSV-N, VSV-P, and VSV-M proteins in cells infected with VSV (Ayala-Breton et al., 2013). Furthermore, other validation studies showed the successful expression of the M protein in SW480 cells treated with wildtype VSV (wtVSV), VSVΔM51 or M51R-M plasmids compared to control cells transfected with empty plasmids (Gray et al., 2019). Another study showed that in cancerous 786–0 cells but not in normal GM-38 cells, infection of colchicine-treated cells with VSVΔM51, wtVSV, or VSVΔM51-GFP increased the expression levels of the viral proteins (Arulanandam et al., 2015). We further characterized the VSVΔ51M virus in a B16F10 subcutaneous treatment model, which we also established in our laboratory. It is unsurprising that the treatment effects were derived from VSVΔ51M and that the parental VSV strain had similar beneficial effects on overall survival, as the results for the virus-treated groups were significantly better than those for the untreated group (Figure 2). This may be because each virus has the same oncolytic capacity, whereas deletion of residue 51M was implemented to reduce toxicity. Furthermore, intratumoral VSV administration increases viral replication in subcutaneously established B16F10 tumors in C57Bl/6 mice. In contrast, *Lemay et al.* demonstrated that intravenous injection of VSVΔM51 (5 × 10^8^ PFU/100 ml) into C57Bl/6 mice bearing subcutaneous B16F10 tumors resulted in a lack of viral replication and low effectiveness of VSV-based therapy (Lemay et al., 2012). This discrepancy may be due to differences in the treatment delivery procedure in which intratumoral therapeutic virus delivery demonstrated better transduction efficiency and therapeutic efficacy than systemic delivery for liver cancer, likely resulting in a better therapeutic outcome in clinical practice in the future (Kim et al., 2012). These results are consistent with those reported by Abdullahi and others, who found that rVSV-GFP delivered via tail vein injection did not prolong survival in orthotopic HCC-bearing mice compared to PBS (Abdullahi et al., 2018). Additionally, three doses of VSV were administered intratumorally to mice bearing B16F10 tumors, and the number of doses could be increased to five or six to improve treatment efficacy (Wongthida et al., 2011).

We and others are further developing the VSVΔ51M platform to enhance its immunostimulatory properties by including different cytokines, such as GM-CSF, IFN-γ, IL-15 and IL-12, in the viral genome to amplify the immune response to achieve better treatment outcomes (Lemay et al., 2012; Stephenson et al., 2012; Bourgeois-Daigneault et al., 2016). Based on previous studies showing the effective oncolytic activity of VSV expressing murine IL12 in the treatment of head and neck carcinoma (Shin et al., 2007), we used our version of the VSVΔ51M platform to express human IL-12, which is recognized to be a master regulator of antitumor immunity. IL-12 has been demonstrated to possess antitumor properties. It initiates cellular responses to tumor cells and directs subsequent adaptive immune responses enhanced by activating dendritic cells and T lymphocytes (Shin et al., 2007; Ghouse et al., 2020). The expression of hIL-12 was validated and measured in MCF-7 and Vero-E6 cells infected with VSVΔ51M-hIL-12. We found that the production of hIL-12 by infected Vero-E6 cells was slightly higher than that by infected MCF-7 cells (Figure 1E). The higher susceptibility of Vero-E6 cells could explain this variation in viral infection, which leads to more hIL-12 being translated and secreted. It is worth mentioning that the human IL-12 protein does not cross react with murine lymphocytes as investigated previously (Schoenhaut et al., 1992).

Tumors are believed to become susceptible to oncolytic viruses during tumor development due to defects in the antiviral response (Ahmed et al., 2010). Therefore, we qualitatively and quantitatively examined the efficacy of recovered viruses against the cancer cell lines MCF-7, A549, and B16F10, as well as the normal fibroblast cell line GM-38. We found that rescued viruses efficiently entered the indicated tumor cells and induced cytotoxicity at 48, 72, and 96 h of infection at MOIs from 1 to 10. The toxic effect of VSV and VSVΔ51M-hIL-12 on cancer cells was higher than that of VSVΔ51M. This result agrees with a previous study that demonstrated the *in vitro* effects of rVSV-wt and rVSV-M51R on human MCF-7 breast cancer cells. That study found that nontumorigenic MCF10A and highly tumorigenic MCF-7 cells were effectively killed and that viability was reduced at 24 and 48 h after infection with rVSV-wt or rM51R-M (Ahmed et al., 2010). As expected, a similar conclusion was reached, showing that the M-mutant VSV killing of MCF-7 cells was not as rapid as that seen in VSV infected cells. Additionally, we observed that the incorporation of hIL-12 into the viral vector increased the toxicity of the vector compared to that of its parental mutant form. Interestingly, the VSVΔ51M-hIL-12 titer was 1-log lower than the parental VSVΔ51M and VSV titers. This might be due to the location of the hIL-12 insertion in VSVΔ51M, which was downstream of the P gene and therefore closer to the viral promoter, which could have impacted the expression of the M and G genes, resulting in decreased levels of viral RNA replication. Regardless, our *ex vivo* observations indicate that adding hIL-12 to VSVΔ51M significantly enhances the activation of NK cells and promotes IFN-γ production, which warrants further investigation in more clinically relevant models to support our future plans for the clinical development of VSVΔ51M-hIL-12.

## Data Availability

The original contributions presented in the study are included in the article/Supplementary Material, further inquiries can be directed to the corresponding authors.
