# Supplementary material for: Construction of VSVΔ51M oncolytic virus expressing human interleukin-12
Source: Front Mol Biosci. 2023 May 15;10:1190669. doi: 10.3389/fmolb.2023.1190669 (PMC10225647; doi:10.3389/fmolb.2023.1190669)
Supplement: Supplementary file 1 [file DataSheet2.PDF]

(A) CPE

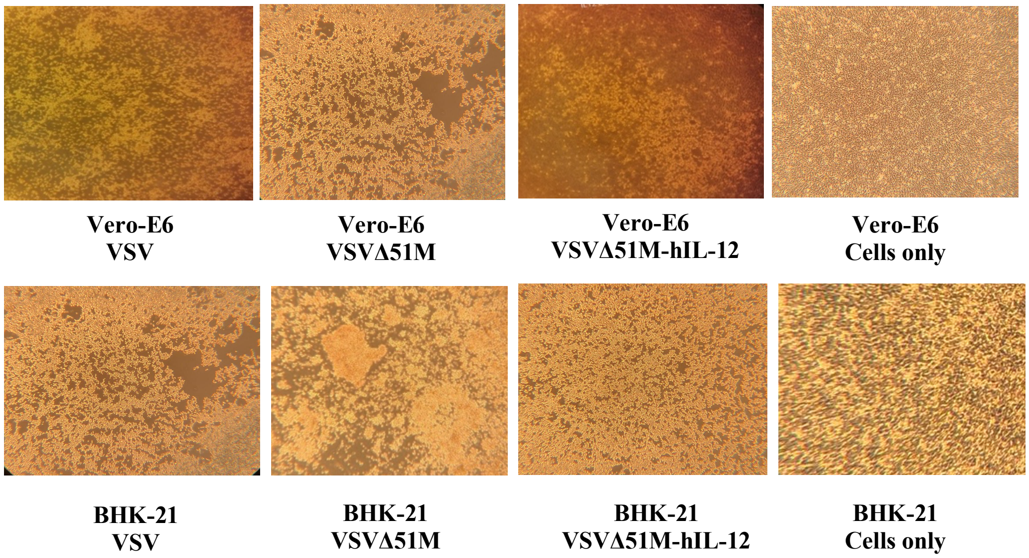

(B) RT-PCR

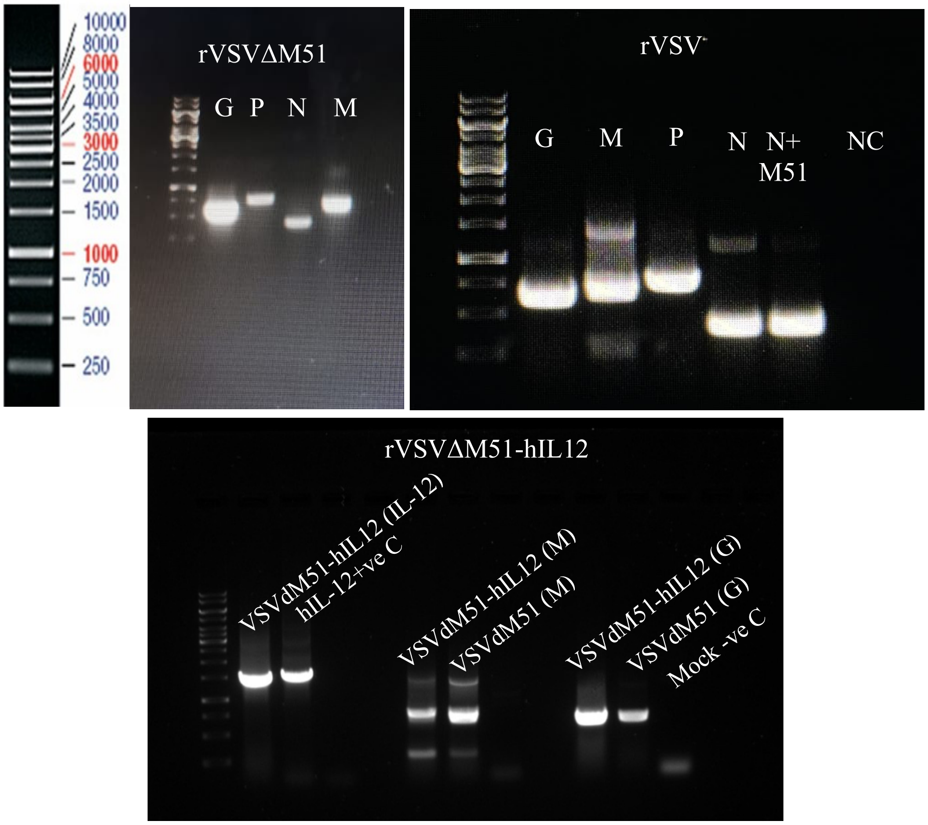

Figure 2: Cytopathic effects and gene expression of VSV viruses (A) CPEs on Vero-E6 and BHK-21 cells infected with recovered VSV viruses. (B) 1% agarose gel electrophoresis of RT–PCR products to determine the gene expression of rescued VSV, VSVΔ51M, and VSVΔ51M-hIL-12 viruses.
