# Supplementary material for: Construction of VSVΔ51M oncolytic virus expressing human interleukin-12
Source: Front Mol Biosci. 2023 May 15;10:1190669. doi: 10.3389/fmolb.2023.1190669 (PMC10225647; doi:10.3389/fmolb.2023.1190669)
Supplement: Supplementary file 2 [file DataSheet3.PDF]

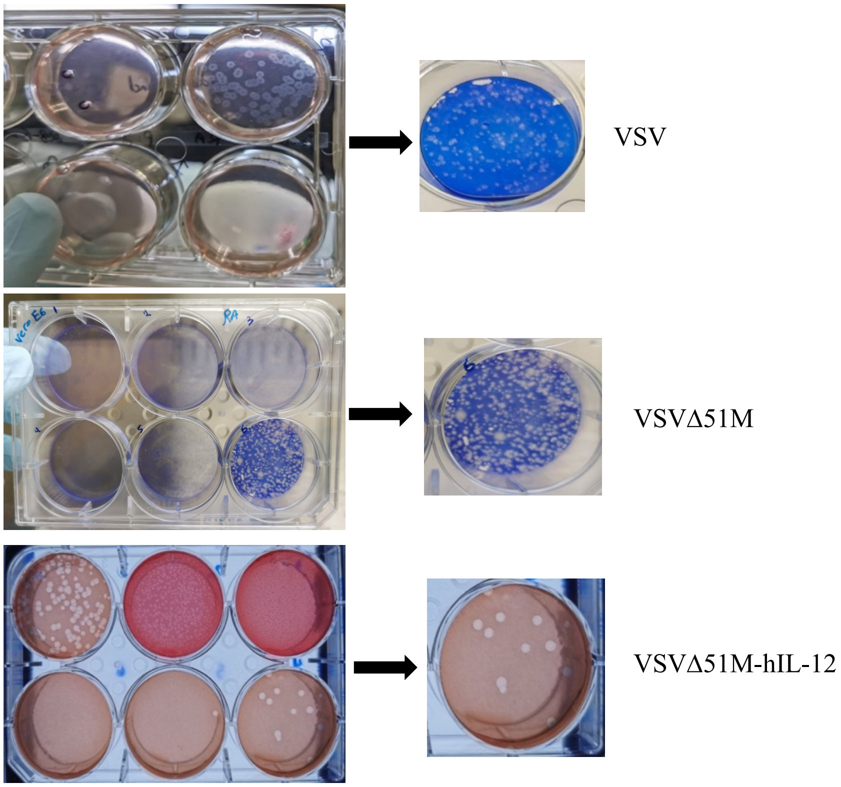

Figure 3: VSVΔ51M and VSVΔ51M-hIL-12 viral plaque assays. Vero-E6 cells were infected with 500 µl of serially diluted virus and incubated for 1 h at 37 °C. After the incubation, 2-ml overlays of 1.6% agarose mixed with 2X complete DMEM were applied to measure viral titers.
