# Supplementary material for: Construction of VSVΔ51M oncolytic virus expressing human interleukin-12
Source: Front Mol Biosci. 2023 May 15;10:1190669. doi: 10.3389/fmolb.2023.1190669 (PMC10225647; doi:10.3389/fmolb.2023.1190669)
Supplement: Supplementary file 3 [file DataSheet1.PDF]

A

B

C

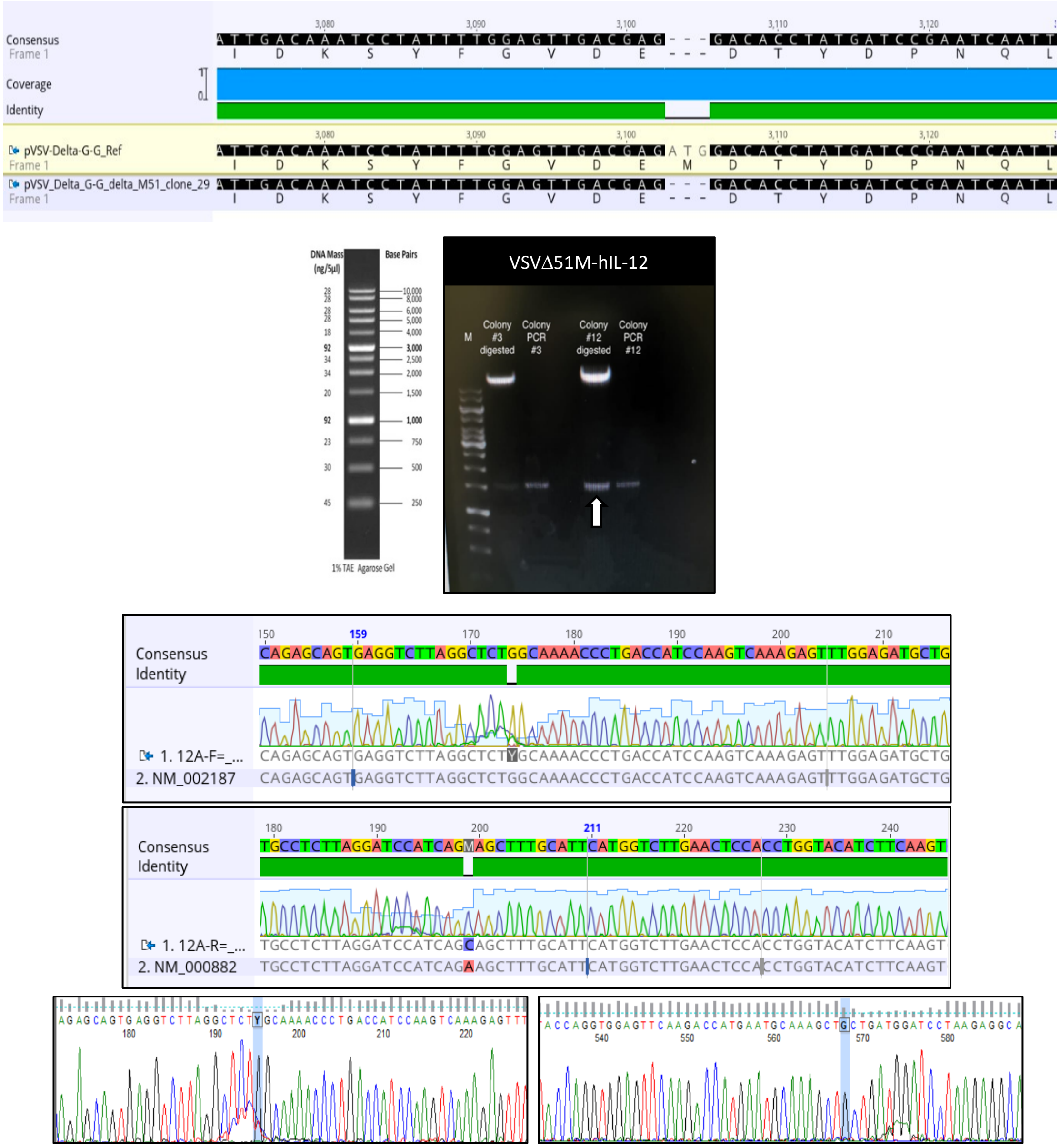

Figure 1: (A) An analysis of the sequence confirmed Δ51M deletion (B) Digestion of VSVΔ51M-hIL-12, visualized by 1% agarose gel electrophoresis. The arrowhead shows the expected size of the *hIL-12* gene insert, which is approximately 1536 bp. The DNA size markers (from top to bottom) correspond to 10,000, 8000, 6000, 5000, 4000, 3000, 2500, 2000, 1500, 1000, 800, 600 and 400 bp. (C) Sequencing analysis of the hIL-12 inserted gene.
